# Hippocampal subfield volumetric changes after radiotherapy for brain metastases

**DOI:** 10.1093/noajnl/vdae040

**Published:** 2024-03-20

**Authors:** Klara Holikova, Iveta Selingerova, Petr Pospisil, Martin Bulik, Ludmila Hynkova, Ivana Kolouskova, Lucie Hnidakova, Petr Burkon, Marek Slavik, Jiri Sana, Tomas Holecek, Jiri Vanicek, Pavel Slampa, Radim Jancalek, Tomas Kazda

**Affiliations:** Department of Medical Imaging, St. Anne’s University Hospital Brno and Faculty of Medicine, Masaryk University, Brno, Czech Republic; Research Center for Applied Molecular Oncology, Masaryk Memorial Cancer Institute, Brno, Czech Republic; Department of Radiation Oncology, Masaryk Memorial Cancer Institute, Brno, Czech Republic; Department of Radiation Oncology, Faculty of Medicine, Masaryk University, Brno, Czech Republic; Department of Medical Imaging, St. Anne’s University Hospital Brno and Faculty of Medicine, Masaryk University, Brno, Czech Republic; Department of Radiation Oncology, Masaryk Memorial Cancer Institute, Brno, Czech Republic; Department of Radiation Oncology, Faculty of Medicine, Masaryk University, Brno, Czech Republic; Department of Comprehensive Cancer Care, Masaryk Memorial Cancer Institute, Brno, Czech Republic; Department of Comprehensive Cancer Care, Faculty of Medicine, Masaryk University, Brno, Czech Republic; Department of Radiation Oncology, Masaryk Memorial Cancer Institute, Brno, Czech Republic; Department of Radiation Oncology, Faculty of Medicine, Masaryk University, Brno, Czech Republic; Department of Radiation Oncology, Masaryk Memorial Cancer Institute, Brno, Czech Republic; Department of Radiation Oncology, Faculty of Medicine, Masaryk University, Brno, Czech Republic; Department of Radiation Oncology, Masaryk Memorial Cancer Institute, Brno, Czech Republic; Department of Radiation Oncology, Faculty of Medicine, Masaryk University, Brno, Czech Republic; Department of Comprehensive Cancer Care, Masaryk Memorial Cancer Institute, Brno, Czech Republic; Central European Institute of Technology, Masaryk University, Brno, Czech Republic; Department of Medical Imaging, St. Anne’s University Hospital Brno and Faculty of Medicine, Masaryk University, Brno, Czech Republic; Research Center for Applied Molecular Oncology, Masaryk Memorial Cancer Institute, Brno, Czech Republic; Department of Biomedical Engineering, Brno University of Technology, Brno, Czech Republic; Department of Medical Imaging, St. Anne’s University Hospital Brno and Faculty of Medicine, Masaryk University, Brno, Czech Republic; Department of Radiation Oncology, Masaryk Memorial Cancer Institute, Brno, Czech Republic; Department of Radiation Oncology, Faculty of Medicine, Masaryk University, Brno, Czech Republic; Department of Neurosurgery, St. Anne’s University Hospital Brno, Brno, Czech Republic; Department of Neurosurgery, St. Anne’s University Hospital Brno, Faculty of Medicine, Masaryk University, Brno, Czech Republic; Department of Radiation Oncology, Masaryk Memorial Cancer Institute, Brno, Czech Republic; Department of Radiation Oncology, Faculty of Medicine, Masaryk University, Brno, Czech Republic

**Keywords:** brain, hippocampus, metastasis, radiotherapy, volumetry

## Abstract

**Background:**

Changes in the hippocampus after brain metastases radiotherapy can significantly impact neurocognitive functions. Numerous studies document hippocampal atrophy correlating with the radiation dose. This study aims to elucidate volumetric changes in patients undergoing whole-brain radiotherapy (WBRT) or targeted stereotactic radiotherapy (SRT) and to explore volumetric changes in the individual subregions of the hippocampus.

**Method:**

Ten patients indicated to WBRT and 18 to SRT underwent brain magnetic resonance before radiotherapy and after 4 months. A structural T1-weighted sequence was used for volumetric analysis, and the software FreeSurfer was employed as the tool for the volumetry evaluation of 19 individual hippocampal subregions.

**Results:**

The volume of the whole hippocampus, segmented by the software, was larger than the volume outlined by the radiation oncologist. No significant differences in volume changes were observed in the right hippocampus. In the left hippocampus, the only subregion with a smaller volume after WBRT was the granular cells and molecular layers of the dentate gyrus (GC-ML-DG) region (median change −5 mm^3^, median volume 137 vs. 135 mm^3^; *P* = .027), the region of the presumed location of neuronal progenitors.

**Conclusions:**

Our study enriches the theory that the loss of neural stem cells is involved in cognitive decline after radiotherapy, contributes to the understanding of cognitive impairment, and advocates for the need for SRT whenever possible to preserve cognitive functions in patients undergoing brain radiotherapy.

**Key Points**

First study on post-RT changes in hippocampal subregions for WBRT versus SRT.
Only the left dentate gyrus showed volume post-WBRT change.
Support theory linking neural stem cell loss to post-RT cognitive decline.

**Importance of the Study**

Our study deepens the understanding of radiation-induced neurocognitive decline, focusing on hippocampal damage. Uniquely, we examine post-radiation changes in hippocampal subregions for patients undergoing WBRT and targeted stereotactic radiotherapy. Notably, the left dentate gyrus in the WBRT group exhibited a volume decrease, unlike the right side or the SRT-treated control group. The dentate gyrus, crucial for neurogenesis and memory, especially in verbal learning, faces unilateral impairment post-WBRT. This highlights the importance of assessing specific changes in radiation-induced hippocampal damage. Our findings support the theoretical basis for unilateral hippocampal impairment and stress the need for targeted strategies to preserve cognitive functions during brain radiotherapy. Further research is vital for comprehending multifactorial changes after radiotherapy and optimizing strategies to enhance overall brain metastases patient quality of life.

Radiotherapy (RT) is routinely employed for treating both limited and multiple brain metastases (BM), with nearly all BM patients being recommended for RT at some point during their disease course.^1^ Nevertheless, cognitive toxicity has been associated with radiation doses to the neuroregenerative zone of the hippocampus (HP) following brain RT. To address this concern, hippocampal avoidance (HA) has been proposed.^2,3^ This involves utilizing intensity-modulated RT during whole-brain radiotherapy (WBRT), hypothesized to preserve cognitive function in patients with multiple BM.^4^ Additionally, stereotactic radiotherapy (SRT) is employed to preserve HP as well as other parts of the brain in patients with a limited number of BM.^5^

Several mechanisms are presumed to be at least partially responsible for post-RT cognitive impairment and hippocampal changes following RT. HP is recognized as one of the most radiosensitive regions of the brain. Changes in HP after radiation can be a primary factor influencing neurocognitive functions, particularly memory, and consequently, the overall quality of life. Numerous studies have reported radiation dose-dependent atrophy of HP after treating brain tumors. Some studies have observed a post-RT reduction in hippocampal volume, while others have subsequently calculated hippocampal volume based on estimated hippocampal age.^6–8^

In routine clinical practice, when conducting HA-WBRT, meeting the required dose-volume constraints in certain HP regions may pose challenges without compromising the treatment of the target volume (resp of the brain). It would be valuable to understand whether specific parts of HP exhibit varying sensitivity or resistance to ionizing radiation. The objective of this secondary exploratory analysis was to elucidate volume changes in our previously published cohort of patients who underwent WBRT and investigate hippocampal changes in another cohort of patients subjected to targeted SRT.^9^ Rigorous hippocampal volumetry was employed to assess post-RT alterations in specific areas of HP.

## Materials and Methods

### Patient Population and Radiotherapy

The study was performed on adult patients with BM outside the hippocampal region enrolled in the prospective study focused on magnetic resonance (MR) spectroscopy changes in HP after RT. This study was approved by the Institutional Ethical Committee (2017/1896/MOU), and all patients provided their written informed consent before study enrollment. This research has been performed in accordance with the Declaration of Helsinki.

The first, previously published, cohort was patients irradiated for BM between May 2013 and February 2015 by WBRT as per indication criteria during that time,^9^ and the second cohort forms patients irradiated for BM between May 2018 and May 2021 by SRT. External beam WBRT was administered using 2 standard opposing lateral fields, which were equally weighted and shaped by a multi-leaf collimator. The radiation therapy (RT) beams were outlined using a 2D simulator (Varian Acuity iX). A uniform prescribed dose of 30 Gy in 10 fractions was delivered over 2 weeks using 6 megavoltage photon beams from a linear accelerator. The whole hippocampi were uniformly irradiated as well.

The SRT plans, administrated in the second cohort, were generated utilizing EclipseTM (Varian Medical Systems, Palo Alto, CA, USA) and executed using TrueBeam STx (Varian Medical Systems, Palo Alto, CA, USA). Noninvasive immobilization was realized by the ORFIT thermoplastic mask and/or CIVCO trUpoint ARCH. For postoperative cases, the cavity was contoured as the gross tumor volume, with a 1–2 mm margin constituting the clinical target volume.

In all patients, we performed both preradiation MR spectroscopic examination focused on HP and the same control MR 4 months after RT. These MRs were used for this planned secondary volumetric analysis to evaluate volumetric changes in hippocampal subregions in cohorts with and without hippocampal irradiation. Thus, patients with SRT (without hippocampal irradiation) serve as a control cohort. Only patients with sufficient quality of both pre-RT as well as follow-up MR were involved in this secondary analysis. In total, hippocampal subregion volume measurement was performed in 10 patients with WBRT (30 Gy in 10 fractions to the whole brain) and 18 subjects with SRT (most commonly 25 Gy in 5 fractions or 24 Gy in 3 fractions).

Inclusion criteria were measurable BM outside a 5-mm margin around either HP on pre-RT MR, age ≥ 18 years, Karnofsky performance status ≥ 70%, and favorable survival prognosis of more than 3.8 months as predicted by the graded prognostic assessment score.^10^ Patients with leptomeningeal disease, a history of neurological or psychiatric disease, patients with hippocampal MR pathology found during pretreatment MR, those with prior RT to the brain, patients suffering from severe active comorbidity affecting the performance of neurocognitive function testing or having a contraindication to MR imaging including severe claustrophobia were excluded. No chemotherapy or other type of systemic treatment was administrated during radiotherapy.

### MR Acquisition

Pre-RT and post-RT MRs were collected for each patient. Structural T1-weighted images were acquired on a GE Discovery 750w 3.0 Tesla unit (General Electric). T1-weighted MR images were acquired with a 3D fast spoiled gradient echo (FSPGR) sequence. The following parameters were used during image acquisition: Echo time (TE) = 4.3 ms, repetition time(TR) = 9.8 ms, flip angle = 12°, matrix: 320 × 224, slice thickness = 0.7 mm, and reconstructed voxel resolution of 0.469 mm.

### Hippocampal Subfield Segmentation

**Figure 1.**
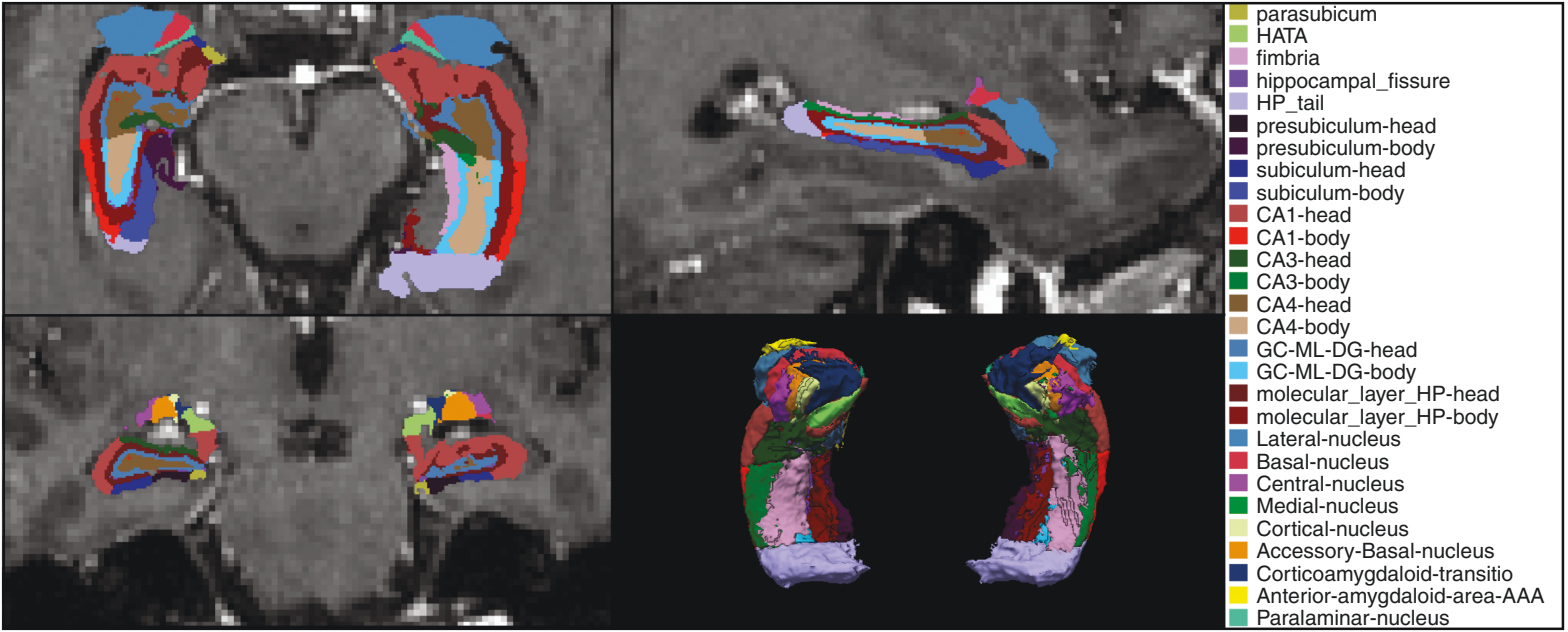
FreeSurfer software version 7.1.1 for semiautomatic segmentation of subhippocampal regions. Amygdala regions were not included as hippocampal subregions. Abbreviations: CA, Cornu Ammonis; GC-ML-DG, granule cell and molecular layer of the dentate gyrus; HATA, hippocampus amygdala transition area; HP, hippocampus

All MR data were initially processed using the standard “recon-all” procedure in version 7.1.1 of the FreeSurfer software. Based on the general segmentation, the hippocampal subfield segmentation for a single subject and a T1 input is started by “segmentHA_T1.sh.” The output consists of several files, one of which is a text file storing estimated volumes of hippocampal substructures and the entire hippocampus.^11^ Segmented parts of hippocampi are listed in Table 1; an example is shown in Figure 1.

**Table 1.** Hippocampal Regions for Subfield Segmentation

Part of the hippocampus	Subregion
Head	Parasubiculum
	Presubiculum-head
	Subiculum-head
	CA1-head
	CA3-head
	CA4-head
	GC-ML-DG-head
	Molecular_layer_HP-head
	HATA
Body	Presubiculum-body
	Subiculum-body
	CA1-body
	CA3-body
	CA4-body
	GC-ML-DG-body
	Molecular_layer_HP-body
	fimbria
Tail	Hippocampal_tail
Fissure	Hippocampal-fissure

Abbreviations: CA, Cornu Ammonis; GC-ML-DG, granule cell and molecular layer of the dentate gyrus; HATA, hippocampus amygdala transition area; HP, hippocampus.

The whole brain was further segmented semiautomatically using EclipseTM (Varian Medical Systems, Palo Alto, CA, USA) software for RT planning. Whole HP was manually segmented by 2 experienced radiation oncologists with reference to RTOG hippocampal contouring online atlas^12^ and reviewed by a neuroradiologist.

### Statistical Analysis

Patient and treatment characteristics were described using the standard summary statistics, ie, median and interquartile range (IQR) for continuous variables and numbers and percentages for categorical variables. Depending on the nature of the data, Fisher’s exact or chi-square test for categorical variables and nonparametric Mann–Whitney test for continuous variables were used to compare differences between the WBRT and SRT groups. Volume pre- and post-RT changes in HP subregions were evaluated using the Wilcoxon paired test. PFS was defined as the time from RT termination to the disease progression or death from any cause. OS was defined as the time from the RT termination to death due to death from any cause. OS and PFS were estimated using the Kaplan–Meier method. A significance level of 5% was considered, and R statistical software version 4.3.1 was used.

## Results

### Patient Characteristics

From 18 patients who underwent WBRT, 10 patients had sufficient quality of both pre-RT and follow-up MR scans for FreeSurfer software analysis. In the control SRT group, sufficient quality of both MR scans had 18 out of 28 patients. A total of 28 patients (median age 63 years, 15 women) were analyzed in this study. The most common primary diagnosis was non-small cell lung cancer (13 patients; 46%), and the majority of patients had a single brain metastasis. Patients in the control SRT group were older (median 64 vs. 60 years; *P* = .029), were in worse overall performance status (median GPA score 3.00 vs. 5.35; *P* < .001), and had a smaller total brain volume (1,298 vs. 1,402 cm^3^; *P* = .018), probably due to a higher proportion of women (67% vs. 30%; *P* = .114). The other basic patient characteristics are summarized in Table 2. During a median follow-up period of 41 months, 24 (86%) patients died. The median PFS was 4.7 and 7.3 months, and the median OS was 13.0 and 11.7 months in the control SRT group and WBRT group, respectively.

**Table 2.** Basic Patient Characteristics. Other Primary Diagnoses Include: Gastrointestinal Cancer, Gynecology Cancer, Melanoma, and Testicular Cancer. Significant *P*-values are in bold.

	Overall *N* = 28	control SRT group *N* = 18	WBRT group *N* = 10	*P*-value
Age				**.029**
Median (IQR)	63 (58, 67)	64 (62, 69)	60 (54, 63)	
Range	45, 85	47, 85	45, 65	
Women	15 (54%)	12 (67%)	3 (30%)	.114
Primary diagnosis				.317
Breast cancer	4 (14%)	3 (17%)	1 (10%)	
NSCLC	13 (46%)	10 (56%)	3 (30%)	
RCC	4 (14%)	1 (5.6%)	3 (30%)	
Other	7 (25%)	4 (22%)	3 (30%)	
KPS				.250
70	3 (11%)	3 (17%)	0 (0%)	
80	6 (21%)	5 (28%)	1 (10%)	
90	16 (57%)	9 (50%)	7 (70%)	
100	3 (11%)	1 (5.6%)	2 (20%)	
Number of BMs				>.999
1	19 (68%)	12 (67%)	7 (70%)	
2	3 (11%)	2 (11%)	1 (10%)	
≥3	6 (21%)	4 (22%)	2 (20%)	
GPA				**<.001**
Median (IQR)	3.00 (2.25, 3.80)	3.00 (2.00, 3.00)	5.35 (3.80, 6.90)	
Range	1.00, 11.00	1.00, 3.50	3.80, 11.00	
Unknown	2	2	0	
Pre-RT surgery	16 (57%)	10 (56%)	6 (60%)	>.999
Post-RT chemotherapy	19 (68%)	12 (67%)	7 (70%)	>.999
TBV (cm^3^)				**.018**
Median (IQR)	1351 (1246, 1421)	1298 (1225, 1411)	1402 (1361, 1455)	
Range	1090, 1726	1090, 1492	1267, 1726	

Abbreviations: SRT, stereotactic radiotherapy; WBRT, whole-brain radiotherapy; IQR, interquartile range; RT, radiotherapy; NSCLC, non-small cell lung cancer; RCC, renal cell carcinoma; KPS, Karnofsky performance status; BM, brain metastases; GPA, graded prognostic assessment; TBV, total brain volume.

### Hippocampal Subfield Volumetry

**Figure 2.**
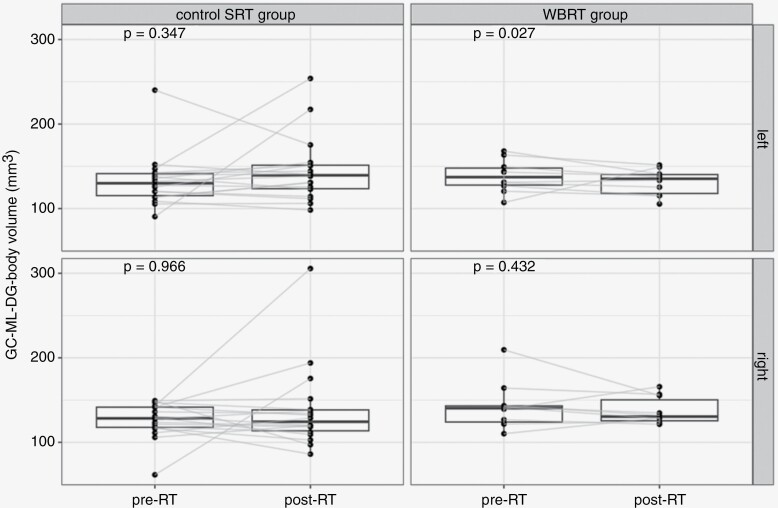
Differences in the volume of left and right GC-ML-DG-body in the control SRT as well as WBRT group. Abbreviations: GC-ML-DG, granule cell and molecular layer of the dentate gyrus; SRT, stereotactic radiotherapy; WBRT, whole-brain radiotherapy; RT, radiotherapy

Volumes of whole HP segmented by FreeSurfer software were significantly larger (*P* < .001) compared to those contoured by radiation oncologists following the RTOG hippocampal contouring atlas (Supplementary Figure 1).^12^ Volume differences between post- and pre-RT MRs in the individual hippocampal subregions for the left and right HP are summarized in Tables 3 and Supplementary Table 1 for the WBRT and control SRT cohort, respectively. No differences in volume changes were observed in the right HP. On the left side, the only subregion with a significantly different volume before and after RT was the GC-ML-DG-body (Figure 2), which turned out to be slightly but consistently smaller (median change −5 mm^3^, median volume 137 vs. 135 mm^3^; *P* = .027).

**Table 3.** Left and Right Hippocampal Subfield Volumes Changes After WBRT. In the „Head“ Section, the HP Indicates the Head of the Whole Hippocampus. Furthermore, Individual Hippocampal Head Subregions are Listed. Significant *P*-values are in bold.

			Left				Right		
		Pre-RT	Post-RT	Change	*P*	Pre-RT	Post-RT	Change	*P*
**Whole HP**					.232				.193
	Median (IQR)	3197 (3108, 3412)	3246 (2981, 3496)	−88 (−193, 30)		3378 (3190, 3565)	3307 (3101, 3685)	−98 (−192, 17)	
	Range	2676, 4335	2602, 4032	−302, 375		3003, 5071	2800, 4386	−817, 1197	
**Head**	**HP**				.770				.232
	Median (IQR)	1,624 (1538, 1675)	1611 (1520, 1849)	−5 (−67, 39)		1676 (1578, 1720)	1651 (1563, 1884)	−27 (−68, 0)	
	Range	1237, 2355	1292, 2203	−177, 257		1460, 2531	1438, 2333	−197, 564	
	**Parasubiculum**				.695				.432
	Median (IQR)	54 (50, 61)	57 (51, 58)	0 (−3, 7)		56 (51, 64)	61 (60, 63)	2 (−3, 9)	
	Range	39, 67	47, 82	−10, 26		47, 76	48, 93	−17, 41	
	**Presubiculum**				.492				.432
	Median (IQR)	129 (120, 138)	131 (117, 148)	2 (−6, 12)		124 (117, 134)	131 (124, 144)	2 (−3, 24)	
	Range	85, 179	107, 163	−17, 22		83, 181	116, 179	−11, 43	
	**Subiculum**				.922				.557
	Median (IQR)	186 (172, 203)	188 (174, 206)	−2 (−8, 12)		180 (169, 199)	184 (174, 217)	1 (−3, 9)	
	Range	124, 268	139, 252	−22, 27		128, 258	159, 254	−24, 50	
	**CA1**				.232				.625
	Median (IQR)	485 (474, 520)	493 (448, 566)	−14 (−36, −3)		499 (457, 549)	509 (463, 584)	−13 (−33, 2)	
	Range	390, 763	380, 695	−67, 102		9, 794	413, 686	−117, 467	
	**CA3**				.770				.846
	Median (IQR)	111 (104, 120)	115 (100, 132)	1 (−3, 10)		123 (103, 134)	116 (106, 132)	4 (−8, 6)	
	Range	90, 153	85, 153	−19, 25		99, 195	102, 184	−32, 36	
	**CA4**				.492				.625
	Median (IQR)	124 (119, 131)	124 (119, 131)	−2 (−8, 6)		127 (119, 137)	127 (121, 144)	−1 (−5, 2)	
	Range	93, 172	93, 172	−16, 12		111, 202	110, 190	−11, 28	
	**GC-ML-DG**				.695				.432
	Median (IQR)	148 (141, 156)	153 (135, 164)	−2 (−9, 7)		155 (144, 166)	152 (140, 169)	−5 (−10, 2)	
	Range	111, 214	115, 206	−18, 16		133, 244	131, 231	−14, 37	
	**Molecular layer HP**				.922				.432
	Median (IQR)	325 (307, 331)	321 (309, 361)	1 (−13, 11)		329 (310, 339)	326 (308, 372)	−2 (−11, 2)	
	Range	239, 474	251, 438	−37, 46		280, 496	278, 459	−38, 105	
	**HATA**				.695				.160
	Median (IQR)	55 (49, 60)	50 (48, 63)	−2 (−3, 5)		65 (60, 70)	56 (54, 67)	−4 (−11, 0)	
	Range	43, 70	41, 76	−23, 16		53, 91	51, 88	−20, 28	
**Body**	**HP**				.432				.770
	Median (IQR)	1104 (1048, 1181)	1110 (1033, 1225)	−45 (−75, 31)		1096 (1071, 1322)	1123 (1049, 1263)	−22 (−61, 50)	
	Range	950, 1407	911, 1314	−98, 128		937, 1726	966, 1584	−526, 514	
	**Presubiculum**				.846				.432
	Median (IQR)	153 (146, 165)	152 (135, 185)	−2 (−12, 19)		151 (123, 184)	154 (135, 174)	4 (−2, 9)	
	Range	134, 177	121, 208	−16, 44		113, 200	117, 217	−70, 99	
	**Subiculum**				.922				.846
	Median (IQR)	226 (211, 235)	226 (209, 239)	1 (−18, 11)		230 (197, 261)	226 (210, 244)	8 (−16, 18)	
	Range	168, 285	191, 257	−29, 27		163, 316	177, 303	−139, 79	
	**CA1**				.232				.105
	Median (IQR)	122 (111, 131)	110 (93, 140)	−12 (−18, −1)		135 (110, 158)	110 (101, 140)	−14 (−35, −9)	
	Range	82, 160	76, 175	−24, 40		94, 215	93, 220	−65, 108	
	**CA3**				.322				.275
	Median (IQR)	81 (75, 94)	79 (73, 92)	−3 (−7, −1)		92 (79, 105)	84 (75, 96)	−6 (−14, 2)	
	Range	69, 115	63, 112	−31, 19		74, 163	69, 135	−65, 33	
	**CA4**				.064				.846
	Median (IQR)	124 (112, 134)	116 (112, 125)	−4 (−9, 1)		122 (109, 131)	117 (112, 128)	−3 (−8, 7)	
	Range	98, 148	95, 138	−27, 7		99, 193	109, 153	−62, 19	
	**GC-ML-DG**				**.027**				.432
	Median (IQR)	137 (128, 148)	135 (118, 140)	−5 (−12, −2)		140 (124, 143)	130 (126, 150)	−6 (−10, 5)	
	Range	107, 168	105, 152	−27, 5		110, 209	121, 166	−54, 26	
	**Molecular layer HP**				.275				.232
	Median (IQR)	204 (188, 223)	203 (194, 229)	−12 (−19, 5)		213 (199, 243)	207 (192, 226)	−10 (−18, 1)	
	Range	172, 265	164, 240	−25, 48		171, 347	174, 305	−102, 111	
	**Fimbria**				.922				.131
	Median (IQR)	72 (66, 83)	77 (61, 88)	0 (−16, 22)		78 (61, 98)	98 (86, 104)	9 (4, 41)	
	Range	59, 105	50, 135	−36, 39		47, 169	75, 140	−84, 55	
**HP tail**					.064				.105
	Median (IQR)	526 (473, 566)	483 (444, 537)	−51 (−71, −17)		591 (524, 638)	531 (503, 565)	−58 (−82, −1)	
	Range	444, 628	399, 559	−90, 79		440, 814	395, 655	−269, 119	
**HP fissure**					.432				.625
	Median (IQR)	152 (106, 164)	141 (130, 164)	1 (−5, 28)		128 (109, 176)	144 (127, 151)	19 (−26, 42)	
	Range	82, 177	97, 217	−30, 47		80, 261	105, 252	−81, 103	

Abbreviations: CA, Cornu Ammonis; GC-ML-DG, granule cell and molecular layer of the dentate gyrus; HATA, hippocampus amygdala transition area; HP, hippocampus; RT, radiotherapy; IQR, interquartile range.

## Discussion

This study aimed to deepen the understanding of radiation damage to HP. RT for BM is associated with alteration of neurocognitive function in virtually all patients, even in patients receiving targeted RT. For example, in a seminal randomized trial comparing targeted SRS with SRS plus WBRT in 1–3 BM, cognitive deterioration at 3 months was less after SRS alone than when combined with WBRT. However, more than half of the SRS patients still suffered from cognitive deterioration (40/63 patients).^5^ Thus, post-RT changes are likely multifactorial, and the overall quality of life and neurocognitive changes will be influenced by factors beyond RT alone.^2,13^ Among the most commonly presumed mechanisms, depletion of neurogenesis within the hippocampal dentate gyrus (compromised neural progenitor cells)^14^ is often discussed. It forms the theoretical basis for the concept of hippocampal avoiding WBRT, which is beneficial, especially for patients with less severe patient-reported cognitive impairment at baseline and those with primary lung histology.^3,15^ Furthermore, inflammation and oxidative stress, vascular damage, white matter changes, neurotransmitter imbalance, synaptic dysfunction, DNA damage, and apoptosis are the supposed mechanisms of post-RT cognitive impairment.^16^ All these mechanisms may contribute to the most commonly affected neurocognitive domain after RT, the verbal memory. Indeed, the test evaluating verbal memory, for example, the Hopkin verbal learning test, is the gold standard for clinical validation of neurocognitive impairment after RT.^17^

To the best of our knowledge, our study is the first to assess post-radiation changes in hippocampal subregions when comparing patients with WBRT and targeted RT to BM. Although there were some differences between the 2 groups of patients, they were comparable from an oncological point of view, especially regarding the spectrum of primary diagnoses (NSCLC) and the number of metastases (median 1 BM in both groups). Both of these baseline characteristics may be those most affecting the assessment of neurocognitive function. These and possibly other biases are further minimized by considering the individual patient itself as a control in this study and assessing the change in hippocampal subvolume size over time (control MR vs. preradiation MR). Surprisingly, no differences in the whole HP volume were observed in our study compared to the study by Seibert et al., who described radiation dose-dependent hippocampal atrophy detected with longitudinal volumetric MR; however, in patients with higher RT dose.^7^ The only subregion that showed a decreased volume was the left granule cell and molecular layer of the dentate gyrus in the WBRT group. On the right side, as well as in the control group of patients receiving SRT (no hippocampal irradiation), the volume was not significantly different in the dentate gyrus as well as in the rest of the HP.

Like the HP, the dentate gyrus consists of three distinct layers:Aan outer molecular layer, a middle granule cell layer, and an inner polymorphic layer. It is involved in forming and processing new memories, particularly implicated in spatial learning and pattern separation.^18,19^ Research on patients who have undergone unilateral medial temporal lobe resection for treating epilepsy suggests that the resection of the left HP impairs verbal memory tasks, specifically affecting learning and retention of story content, word recognition, recall, and verbal associative memory.^20,21^ In contrast, resections of the right HP and parahippocampal cortex lead to deficits in visuospatial tasks.^22^ Patients undergoing right temporal lobectomy exhibit impaired topographical memory, specifically affecting their navigation, scene recognition, and ability to draw maps from memory. Patients with left temporal lobectomy experience difficulties in context-dependent episodic memory, manifesting as impaired memory for specific events.^23^

A study similar to ours focused on comparing hippocampal atrophy after WBRT and HA-WBRT, finding that atrophy after HA-WBRT is 3 times lower. However, our study delves deeper into the issue, not only focusing on the entire HP but also its individual parts.^6–8^ The observation of only left hippocampal subfield volume change after WBRT, more importantly only in the gyrus dentatus subfield region, further strengthens the theoretical basis for unilateral hippocampal radiotherapy injury.^24^ The NCT04801342 study entitled “Neurocognitive Outcome of Bilateral or Unilateral HA WBRT With Memantine for BM” is the ongoing and recruiting clinical trial evaluating the potential of unilateral hippocampal sparing building on the presumption of only unilateral hippocampal post-RT changes being responsible for neurocognitive decline.^25^ In addition to the WBRT and HA-WBRT techniques mentioned above, targeted stereotactic irradiation is increasingly employed in patients with multiple metastases.^26,27^ It is reasonable to assume that in cases where metastases are not proximal to the hippocampi, the volume changes in different hippocampal subfields would be less pronounced compared to HA-WBRT. However, even with targeted radiotherapy, there is increased cerebral low-dose bath with an increasing number of metastases, potentially contributing to the progressive decline in cognitive functions, possibly also through mechanisms involving hippocampal alterations. Further studies are warranted to assess the safe threshold for the number and size of multiple metastases suitable for stereotactic radiotherapy. In a recent in silico study, Becker et al. estimated that stereotactic radiosurgery of 12–13 tumors per day over 10 days would deliver the dose of radiation to healthy brain tissue typically associated with a standard course of WBRT.^28^ On the other hand, in another recent retrospective study on 184 patients treated for 915 BM by repeated postoperative SRS or STT recurrent BM, even in patients who were treated for more than 10 BM, the median volume equivalent to the WBRT dose remained low.^29^

From several available automated segmentation tools, we have chosen FreeSurfer for our study. This tool is widely accessible, well-automated, well-documented, actively developed, and easily implementable. Previous studies suggest that earlier versions of FreeSurfer approach the results of manual methods more closely than other common tools, such as FSL.^30,31^ While there are some classification systems specific to the hippocampus and automated brain segmentation systems that have outperformed FreeSurfer in precise hippocampal segmentations, FreeSurfer has demonstrated greater sensitivity to atrophy.^30,32–34^ Independent scans of the same individual show consistent, albeit imperfect, agreement in results from analyses conducted using FreeSurfer.^35^ These advantages, along with its ongoing active development, make it our preferred choice for automated hippocampal segmentation. Version 7.1.1 reduces discrepancies in volume analyses and volume distortions.

## Conclusions

In conclusion, our study delves into the nuanced impact of RT on hippocampal subregions in patients with BMs. The observed decrease in the left granule cell and molecular layer of the dentate gyrus after WBRT underscores the importance of considering specific subfield changes in the assessment of hippocampal radiation injury. This finding aligns with the theoretical basis for unilateral hippocampal radiotherapy injury, as supported by the ongoing clinical trial exploring the potential benefits of unilateral hippocampal sparing. Our research contributes to the evolving understanding of RT-induced cognitive impairment, emphasizing the need for targeted approaches to preserve cognitive function in patients undergoing brain RT. Further investigations, considering the multifactorial nature of post-RT changes, will be crucial to inform strategies that enhance patients’ overall quality of life and our results may enhance identification of potential predictive biomarkers for advanced radiotherapy cognitive sparing strategies.

## Supplementary Material

vdae040_suppl_Supplementary_Data
