# Supplementary material for: Hippocampal subfield volumetric changes after radiotherapy for brain metastases
Source: Neurooncol Adv. 2024 Mar 20;6(1):vdae040. doi: 10.1093/noajnl/vdae040 (PMC11032105; doi:10.1093/noajnl/vdae040)
Supplement: vdae040_suppl_Supplementary_Data [file vdae040_suppl_supplementary_data.zip › Supplementary Figure Legends.docx]

Supplementary Figure 1: differences in hippocampal volumes between FreeSurfer software-based and radiation oncologists (RTOG hippocampal contouring atlas)-based segmentation. Abbreviations: SRT, stereotactic radiotherapy; WBRT, whole brain radiotherapy
