# Supplementary material for: Hippocampal subfield volumetric changes after radiotherapy for brain metastases
Source: Neurooncol Adv. 2024 Mar 20;6(1):vdae040. doi: 10.1093/noajnl/vdae040 (PMC11032105; doi:10.1093/noajnl/vdae040)
Supplement: vdae040_suppl_Supplementary_Data [file vdae040_suppl_supplementary_data.zip › Supplementary Table.docx]

Supplementary table 1: Left and right hippocampal subfield volumes changes after SRT. In the „Head“ section, the HP indicates the head of the whole hippocampus. Further, individual hippocampal head subregions are listed.

|  |  |  | **left** |  |  |  | **right** |  |  |
| --- | --- | --- | --- | --- | --- | --- | --- | --- | --- |
|  |  | **pre-RT** | **post-RT** | **change** | **p** | **pre-RT** | **post-RT** | **change** | **p** |
| **Whole HP** | |  |  |  | 0.609 |  |  |  | 0.766 |
|  | Median (IQR) | 3,213  (2,956, 3,461) | 3,289  (2,998, 3,851) | -23  (-109, 51) |  | 3,291  (3,160, 3,525) | 3,189  (2,999, 3,763) | -86  (-288, 379) |  |
|  | Range | 1,974, 5,878 | 2,402, 5,271 | -1,905, 3,090 |  | 1,620, 4,457 | 1,891, 6,693 | -1,482, 2,712 |  |
| **Head** | **HP** |  |  |  | 0.246 |  |  |  | 0.832 |
|  | Median (IQR) | 1,565  (1,491, 1,771) | 1,603  (1,421, 1,859) | -63  (-115, -16) |  | 1,620  (1,553, 1,739) | 1,518  (1,434, 1,848) | -1  (-138, 164) |  |
|  | Range | 902, 2,852 | 1,115, 2,604 | -1,097, 1,551 |  | 746, 2,366 | 833, 2,832 | -786, 927 |  |
|  | **Parasubiculum** | |  |  | 0.609 |  |  |  | 0.640 |
|  | Median (IQR) | 55 (48, 61) | 53 (48, 67) | -1 (-9, 6) |  | 61 (54, 72) | 58 (50, 66) | -2 (-6, 6) |  |
|  | Range | 35, 81 | 33, 84 | -24, 19 |  | 34, 97 | 22, 100 | -34, 32 |  |
|  | **Presubiculum** |  |  |  | 0.671 |  |  |  | 0.766 |
|  | Median (IQR) | 116 (100, 138) | 120 (101, 140) | -3 (-17, 6) |  | 123 (111, 137) | 115 (103, 140) | 1 (-12, 21) |  |
|  | Range | 80, 204 | 67, 206 | -63, 78 |  | 65, 175 | 46, 210 | -77, 67 |  |
|  | **Subiculum** |  |  |  | 0.347 |  |  |  | 0.523 |
|  | Median (IQR) | 156 (144, 188) | 160 (144, 195) | -8 (-20, 7) |  | 169 (158, 192) | 158 (147, 201) | 8 (-15, 30) |  |
|  | Range | 116, 354 | 122, 333 | -158, 169 |  | 78, 257 | 106, 318 | -86, 84 |  |
|  | **CA1** |  |  |  | 0.468 |  |  |  | 0.966 |
|  | Median (IQR) | 502 (458, 570) | 525 (451, 580) | -22 (-35, 13) |  | 503 (482, 546) | 488 (458, 572) | -5 (-50, 38) |  |
|  | Range | 287, 865 | 420, 768 | -319, 481 |  | 252, 775 | 281, 909 | -243, 248 |  |
|  | **CA3** |  |  |  | 0.865 |  |  |  | 0.799 |
|  | Median (IQR) | 120 (105, 126) | 114 (104, 132) | -1 (-9, 12) |  | 126 (104, 136) | 114 (102, 142) | -1 (-18, 13) |  |
|  | Range | 49, 208 | 60, 199 | -92, 150 |  | 46, 147 | 48, 218 | -66, 94 |  |
|  | **CA4** |  |  |  | 0.932 |  |  |  | 0.932 |
|  | Median (IQR) | 120 (105, 127) | 123 (111, 134) | -2 (-9, 8) |  | 127 (109, 136) | 123 (106, 139) | 0 (-13, 15) |  |
|  | Range | 52, 233 | 67, 203 | -112, 143 |  | 41, 147 | 49, 222 | -66, 103 |  |
|  | **GC-ML-DG** |  |  |  | 0.580 |  |  |  | 0.932 |
|  | Median (IQR) | 146 (130, 157) | 149 (132, 164) | -4 (-12, 8) |  | 155 (139, 164) | 147 (128, 171) | 0 (-17, 11) |  |
|  | Range | 67, 274 | 82, 246 | -125, 168 |  | 57, 198 | 68, 267 | -73, 105 |  |
|  | **Molecular layer HP** | |  |  | 0.246 |  |  |  | 0.865 |
|  | Median (IQR) | 298 (289, 341) | 311 (277, 358) | -12 (-24, 1) |  | 322 (301, 338) | 299 (279, 356) | -5 (-31, 32) |  |
|  | Range | 177, 576 | 212, 520 | -225, 315 |  | 139, 467 | 170, 564 | -154, 183 |  |
|  | **HATA** |  |  |  | 0.580 |  |  |  | 0.865 |
|  | Median (IQR) | 56 (53, 64) | 60 (53, 67) | -2 (-5, 3) |  | 62 (54, 68) | 60 (49, 68) | 1 (-11, 11) |  |
|  | Range | 38, 89 | 49, 82 | -13, 44 |  | 33, 88 | 40, 107 | -26, 22 |  |
| **Body** | **HP** |  |  |  | 0.702 |  |  |  | 0.799 |
|  | Median (IQR) | 1,133  (1,042, 1,210) | 1,203  (1,074, 1,263) | -3  (-41, 69) |  | 1,130  (1,080, 1,243) | 1,128  (1,005, 1,259) | -21  (-97, 136) |  |
|  | Range | 822, 2,180 | 890, 2,233 | -511, 1,063 |  | 538, 1,322 | 780, 2,708 | -395, 1,447 |  |
|  | **Presubiculum** |  |  |  | 0.154 |  |  |  | 0.671 |
|  | Median (IQR) | 154 (138, 177) | 167 (145, 187) | 7 (-7, 23) |  | 141 (137, 157) | 144 (124, 172) | 0 (-14, 25) |  |
|  | Range | 93, 289 | 93, 276 | -113, 107 |  | 62, 169 | 104, 353 | -53, 204 |  |
|  | **Subiculum** |  |  |  | 0.766 |  |  |  | 0.640 |
|  | Median (IQR) | 211 (190, 232) | 226 (201, 293) | 1 (-15, 22) |  | 222 (185, 232) | 212 (183, 244) | 11 (-21, 19) |  |
|  | Range | 173, 427 | 154, 411 | -119, 202 |  | 95, 265 | 154, 496 | -101, 273 |  |
|  | **CA1** |  |  |  | 0.832 |  |  |  | 0.495 |
|  | Median (IQR) | 124 (109, 154) | 127 (116, 178) | 0 (-14, 12) |  | 132 (118, 137) | 132 (112, 156) | 3 (-18, 22) |  |
|  | Range | 69, 273 | 83, 261 | -93, 177 |  | 61, 165 | 91, 307 | -48, 142 |  |
|  | **CA3** |  |  |  | 0.181 |  |  |  | 0.899 |
|  | Median (IQR) | 88 (78, 91) | 93 (80, 105) | 5 (-2, 7) |  | 92 (88, 96) | 88 (83, 103) | -4 (-9, 13) |  |
|  | Range | 45, 162 | 61, 202 | -16, 88 |  | 47, 104 | 56, 236 | -22, 141 |  |
|  | **CA4** |  |  |  | 0.325 |  |  |  | 0.966 |
|  | Median (IQR) | 118 (103, 132) | 126 (112, 140) | 2 (-6, 18) |  | 121 (105, 127) | 115 (102, 131) | -4 (-11, 13) |  |
|  | Range | 80, 222 | 89, 235 | -66, 109 |  | 57, 139 | 77, 281 | -50, 158 |  |
|  | **GC-ML-DG** |  |  |  | 0.347 |  |  |  | 0.966 |
|  | Median (IQR) | 130 (115, 141) | 139 (124, 151) | 2 (-8, 17) |  | 128 (118, 142) | 124 (114, 138) | -3 (-10, 13) |  |
|  | Range | 91, 240 | 98, 254 | -65, 127 |  | 62, 149 | 86, 306 | -51, 163 |  |
|  | **Molecular layer HP** | |  |  | 0.932 |  |  |  | 0.734 |
|  | Median (IQR) | 216 (200, 228) | 225 (201, 242) | -2 (-10, 13) |  | 213 (202, 232) | 213 (192, 245) | 2 (-19, 26) |  |
|  | Range | 154, 425 | 164, 431 | -115, 212 |  | 98, 247 | 150, 525 | -71, 285 |  |
|  | **Fimbria** |  |  |  | 0.212 |  |  |  | 0.734 |
|  | Median (IQR) | 81 (60, 96) | 83 (64, 110) | 3 (-4, 23) |  | 93 (82, 112) | 90 (64, 114) | -4 (-17, 12) |  |
|  | Range | 32, 142 | 44, 210 | -33, 75 |  | 42, 136 | 45, 205 | -50, 87 |  |
| **HP tail** | |  |  |  | 0.196 |  |  |  | 0.609 |
|  | Median (IQR) | 511 (450, 594) | 531 (470, 595) | 13 (-16, 47) |  | 552 (491, 622) | 573 (463, 643) | 15 (-58, 60) |  |
|  | Range | 250, 846 | 397, 1,122 | -297, 477 |  | 337, 769 | 278, 1,153 | -307, 513 |  |
| **HP fissure** | |  |  |  | 0.799 |  |  |  | 0.832 |
|  | Median (IQR) | 129 (125, 156) | 134 (127, 148) | -2 (-20, 23) |  | 137 (117, 160) | 143 (117, 154) | -3 (-8, 17) |  |
|  | Range | 100, 193 | 117, 227 | -46, 127 |  | 108, 192 | 79, 200 | -97, 56 |  |

Abbreviations: CA, Cornu Ammonis; GC-ML-DG, granule cell and molecular layer of the dentate gyrus; HATA, hippocampus amygdala transition area; HP, hippocampus; RT, radiotherapy; IQR, interquartile range
