# Supplementary figures and images for: Hippocampal subfield volumetric changes after radiotherapy for brain metastases
Source: Neurooncol Adv. 2024 Mar 20;6(1):vdae040. doi: 10.1093/noajnl/vdae040 (PMC11032105; doi:10.1093/noajnl/vdae040)

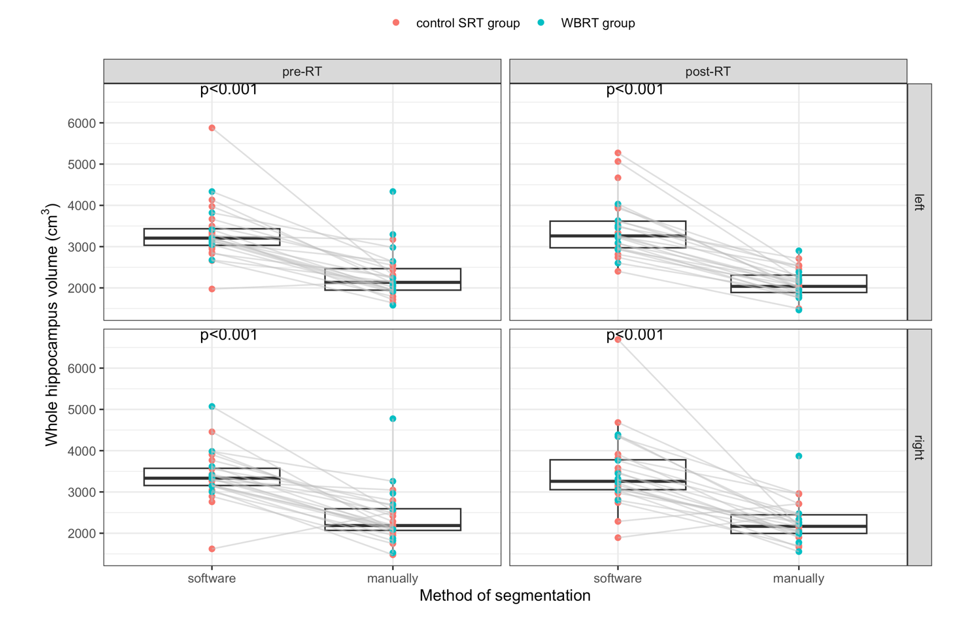

Supplement: vdae040_suppl_Supplementary_Data [file vdae040_suppl_supplementary_data.zip › Supplementary Figure 1.tif]
